# Immune Targeting of Tetraspanins Involved in Cell Invasion and Metastasis

**DOI:** 10.3389/fimmu.2018.01277

**Published:** 2018-06-12

**Authors:** Felipe Vences-Catalán, Shoshana Levy

**Affiliations:** Division of Oncology, Department of Medicine, Stanford University School of Medicine, Stanford, CA, United States

**Keywords:** cancer, immunotherapy, monoclonal antibodies, bench, bedside

## Abstract

Metastasis is the ultimate consequence of cancer progression and the cause of patients’ death across different cancer types. Patients with initial diagnosis of distant disease have a worst 5-year survival compared to patients with localized disease. Therapies that target primary tumors fail to eradicate distant dissemination of cancer. Recently, immunotherapies have improved the survival of patients with metastatic disease, such as melanoma and lung cancer. However, only a fraction of patients responds to immunotherapy modalities that target the host immune system. The need to identify new druggable targets that inhibit or prevent metastasis is, therefore, much needed. Tetraspanins have emerged as key players in regulating cell migration, invasion, and metastasis. By serving as molecular adaptors that cluster adhesion receptors, signaling molecules, and cell surface receptors; tetraspanins are involved in all steps of the metastatic cascade. They regulate cell proliferation, participate in EMT transition, modulate integrin-mediated cell adhesion, and participate in angiogenesis and invasion processes. Tetraspanins have also been shown to modulate metastasis indirectly through exosomes and by regulating cellular interactions in the immune system. Importantly, targeting individual tetraspanin with antibodies has impacted tumor progression. This review will focus on the contribution of tetraspanins to the metastatic process and their potential as therapeutic tumor targets.

## Introduction

Malignant transformation of healthy tissues gives rise to cancer, this disease affects millions of people worldwide. Moreover, metastases; the dissemination of cancer cells is still the greatest cause of death. Patients diagnosed with localized disease have a better 5-year survival than patients with distant disease at the time of diagnosis (1). Therefore, treatments that prevent or diminish metastatic lesions are much needed, such as identifying new druggable targets involved in the metastatic cascade.

Monoclonal antibodies (mAbs) are the preferred immunotherapeutic tools to either target the host immune system or to target the tumor (2). The most common tumor targets are cell surface molecules, such as EGFR, HER2, Mesothelin, CD19, or CD20 whose cell membrane localization, and sometimes tumor-specific expression, or overexpression in comparison to healthy tissues, makes them suitable for antibody-based therapy (2). More recent approaches to immunotherapy of cancer do not target antigens expressed on tumor cells—they unleash the host immune checkpoint blockade, and have improved the survival of patients with metastatic cancers (3).

Tetraspanins are a family of conserved proteins in eukaryotic cells that spans the membrane four times. In humans, 33 members have been identified with different tissue distribution. Expression of some tetraspanins such as CD37 and CD53 is restricted to hematopoietic cells, whereas others, such as CD9, CD81, and CD151 are more broadly expressed. Tetraspanins serve as membrane scaffolds that bring together surface molecules, such as integrins and cell-specific receptors, additional growing evidence shows that engagement of tetraspanins leads to recruiting signaling molecules thereby activating downstream pathways (4). This plethora of interacting partners allows tetraspanins to function in different cellular processes under physiological conditions but also in disease. Multiple studies have shown that tetraspanins regulate tumor growth, cell adhesion, invasion, and migration of tumor cells, reviewed in Ref. (5, 6). Importantly, targeting some tetraspanins with mAbs has proven to be efficient in eliminating tumor cells and in preventing metastasis in preclinical models (7). Here, we will give an overview of tetraspanins as prognostic markers in tumors, their role in invasion and metastasis, and discuss recent studies aimed at antibody-based targeting of these molecules in cancer.

## Tetraspanins as Prognostic Markers of Cancer Progression

Among the human tetraspanins, Tspan8 and 12, CD9, CD37, CD63, CD81, CD82, and CD151 play a role in cancer progression (5, 6). Down or upregulation of these tetraspanins on tumors has been correlated with either good or bad prognosis in different types of cancers. Historically, several tetraspanins were identified by studies that compared normal and malignant tissues. Tspan8 was originally identified as a tumor-associated antigen by an antibody (CO-029) (8), CD63 by a melanoma-associated antigen (ME491) (9), CD151 was re-identified by an antimetastatic antibody (10), and CD82/KAI1 as a metastasis suppressor gene (11).

More recent studies have demonstrated that loss of CD82/KAI1 expression is correlated with poor prognosis of several cancers, reviewed in Ref. (12). Interestingly, presence of a specific splice variant of CD82 that removes exon 7 increases tumor progression and invasion (13). Similarly, loss of CD37 expression in patients with diffuse large B-cell lymphoma showed significant correlation with decreased survival after R-CHOP therapy (14). And lack of CD37 in mice increased the development of germinal center derived B cell lymphomas (15).

By contrast, CD151 is expressed in different types of cancer and high expression correlates with poor prognosis (16). It is of note that expression of CD151 in the host contributes to cancer progression—CD151 knockout (KO) mice have fewer skin, melanoma, lung, and prostate cancers than their wild type (WT) counterparts (17–20). Similarly, upregulated CD81 expression in melanoma was found to contribute to an enhanced metastatic phenotype (21, 22). Additionally, expression of CD81 in the host contributes to tumor progression; CD81KO mice have fewer metastases of breast and lung tumors in syngeneic mouse models (23). Importantly, expression of CD81 in immune suppressive cells contributes to tumor progression (24). A recent study showed that expression of CD151 in human is associated with a hyper-proliferative T cell phenotype (25), it would be interesting to know if CD151 expressed in mouse immune cells plays a role in tumor progression. Tspan8 is another tetraspanin whose upregulation is correlated with ovarian cancer progression (26). More recently, the presence of Tspan8 mRNA in the blood was shown to be a sensitive marker for colorectal cancer detection (27). Reduced CD9 expression has been correlated with poor prognosis in several types of cancers, including melanoma, lung, breast, colon, prostate, pancreatic ovarian, and prostate, reviewed in Ref. (28). However, this is not the case for esophageal squamous cell carcinomas that express higher CD9 levels than normal esophageal tissues (29). Lack of CD9 in mice that develop spontaneous prostate cancer mirrors the former human studies, namely, CD9 deficiency increased liver metastases, although it had no effect on onset of primary tumors, nor on lung metastases (30).

Thus, tetraspanins are important players during cancer progression, some tetraspanins are upregulated in certain cancer types while others are downregulated. Clearly, tetraspanins have been used as prognostic markers in cancer.

## Interactions of Tetraspanins with Partner Proteins Regulate Invasion and Metastasis

**Figure 1:**
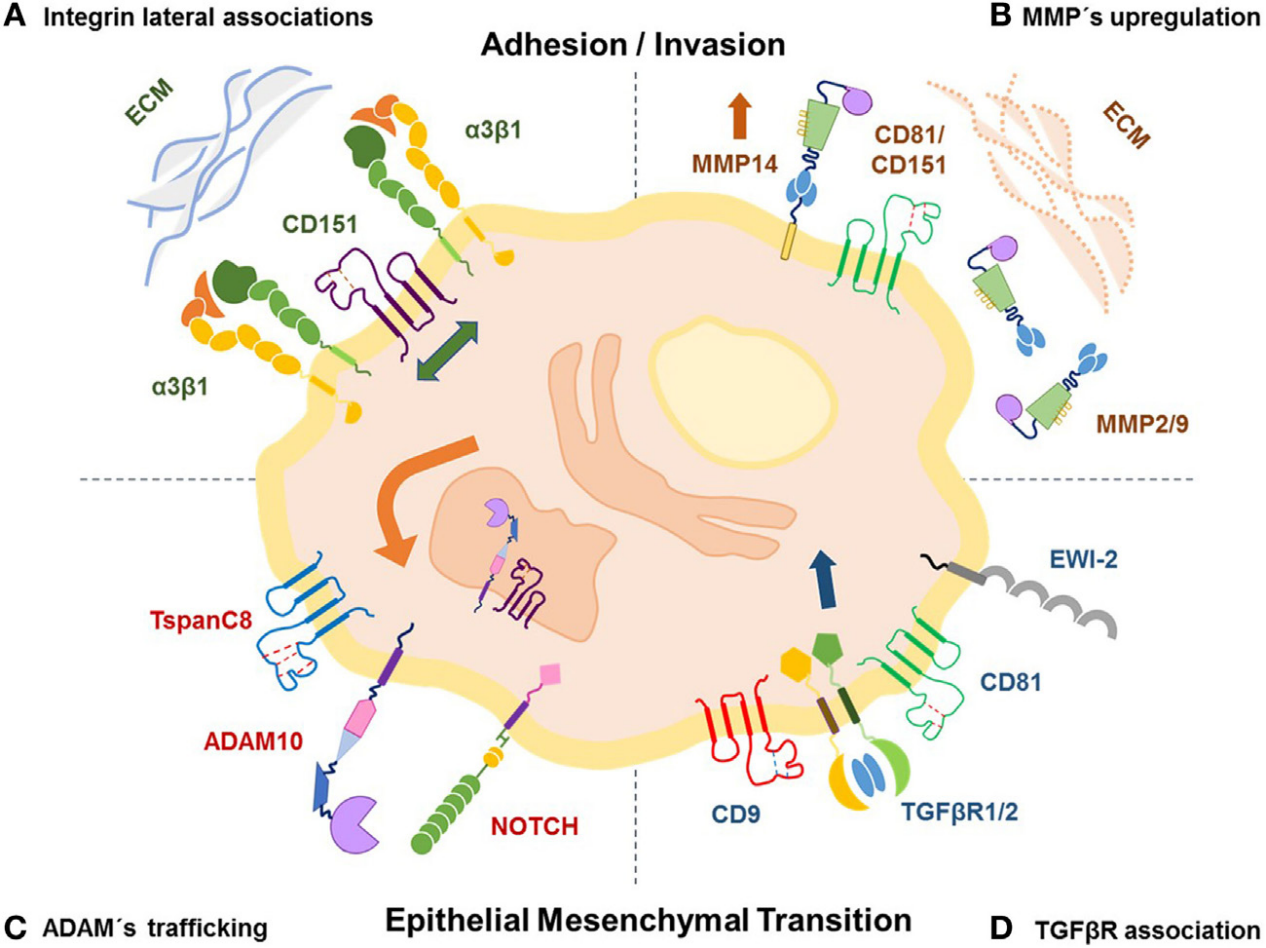
Tetraspanins modulate invasion and metastases by regulating the activity of their partners. **(A)** Tetraspanins form stable lateral association with integrins in tetraspanin-enriched microdomains (TEMS) favoring spreading, cell adhesion, and migration through the extracellular matrix (ECM); on the other hand, **(B)** tetraspanins regulate ECM degradation during cell invasion by modulating expression and activity of metalloproteases, such as MMP-2, MMP-9, and MMP-14. **(C)** The TspanC8 subgroup is known to promote trafficking and cell localization of ADAM10 and its sheddase activity thereby regulating substrates, such as NOTCH receptor, to favor epithelial to mesenchymal transition (EMT). **(D)** Association of CD9/CD81 with EWI-2 affects TGFβ signaling modulating EMT, invasion, and metastases of melanoma.

We now know that tetraspanin-enriched microdomains (TEMs) incorporate partner proteins, such as integrins, cell surface receptors, and metalloproteases (MMPs) that contribute to cellular invasion and metastasis (Figure 1). Biochemically, very few of these complexes are held together when solubilized by harsh detergents, the majority only withstand mild detergents (31). The association of CD151 with laminin-binding integrins α3β1, α6β1, and α6β4 (32) is strong, stoichiometric, and occurs early in biosynthesis (33). Silencing CD151 in epithelial carcinoma cells disrupts α3β1 association with TEMS and impairs cell migration (34). In addition, CD151 ablation reduces cell migration, invasion, spreading, and signaling in an integrin-dependent manner (35). CD9/CD81 also regulate α3β1 integrin, loss of these two tetraspanins impairs breast cancer spreading, motility, and disrupts its association with PKCα in a CD151-independent manner (36). Tspan8 was also shown to modulate invasion of melanoma *in vitro* and *in vivo* through a β1 integrin by affecting integrin-linked kinase signaling and its downstream target AKT (37).

While integrins serve as receptors to extracellular matrix (ECM) components, matrix metalloproteinases (MMPs) degrade ECM components. Several tetraspanins associate with MMP-14 (MT1-MMP) during biosynthesis and prevent its degradation enabling cell surface expression. And this association also involves another known tetraspanin partner, EWI-2 (38). However, a knockdown of a single tetraspanin, such as CD9 or CD81 had no effect on MMP-14-dependent fibronectin degradation, but a CD9/CD81 double knockdown clearly affected degradation (39). Interestingly, overexpression of CD81 in a human melanoma cell line upregulated MT1-MMP expression and activity with a consequent increased invasion and metastases *in vitro* and *in vivo* (21). Similarly, overexpression of CD9 on fibrosarcoma cells increased MMP-9 production and activity, resulting in a more invasive phenotype *in vitro* (40); however, transfection of CD9 into small cell lung cancer cells inhibited transcription of MMP-2 and MMP-14 (41). CD151 was shown to be a link between MMP-14 and integrin α3β1 (42); and to mediate tumor progression by affecting expression and function of MMP-9 in hepatocellular carcinoma (43), melanoma (44), and pancreatic adeno carcinoma (43, 45). Finally, silencing CD63 reduced the levels of β-catenin protein and its downstream target MMP-2 inhibiting metastatic lung colonization of ovarian and melanoma tumors (46).

A disintegrin and metalloproteases (ADAMs) also interact with tetraspanins (47). ADAMs are a family of proteases, classified as sheddases because they can cleave extracellular portions of transmembrane proteins regulating cell functions such as cell invasion and motility. The tetraspanin subgroup TspanC8 (Tspan 5, 10, 14, 15, 17, and 33) mediates trafficking, maturation, and compartmentalization of ADAM10, thereby influencing its function (48, 49). Interestingly, TspanC8 members expressed in *Drosophila* and *C. elegans* were found to regulate ADAM10 levels and to modulate Notch functions that promote epithelial to mesenchymal transition (EMT) in cancer cells (50, 51). Another tetraspanin family member, Tetraspanin-8, is also linked to cancer progression by inducing ADAM12 upregulation in esophageal carcinoma promoting metastases (52).

Transforming growth factor β (TGF-β1) is also regulated by specific tetraspanins. It was shown that absence of CD151 in breast cancer cells affected the compartmentalization of TGFβ receptor 1 thereby disturbing TGF-β1-induced activation of p38, which correlated with reduced lung adhesion and decreased metastases (53). Similarly, CD9/CD81 were shown to regulate TGF-β1 signaling in melanoma by providing critical support for TGFβR2- TGFβR1 association, which in turns favors EMT-like changes, invasion, and metastases (22). However, TGF-β1 signaling is negatively regulated by EWI-motif containing protein 2 (EWI-2), which when present, sequesters CD9/CD81 thereby dissociating EWI-2 interaction with TGF receptors (22). Indeed, lack of EWI-2 in melanoma cells was associated with increased invasion and metastasis *in vitro* and *in vivo* (12).

Tetraspanins clearly regulate tumor progression at different levels by interacting with a plethora of partners, which are implicated in tumor initiation, promotion of an EMT phenotype, invasion, and migration that ultimately lead to metastasis. To better understand the mechanisms of how individual tetraspanin members and their partners contribute to cancer progression *in vivo*, numerous *in vitro* studies have used specific anti-tetraspanins mAbs.

## Targeting Tetraspanins with mAbs

**Figure 2:**
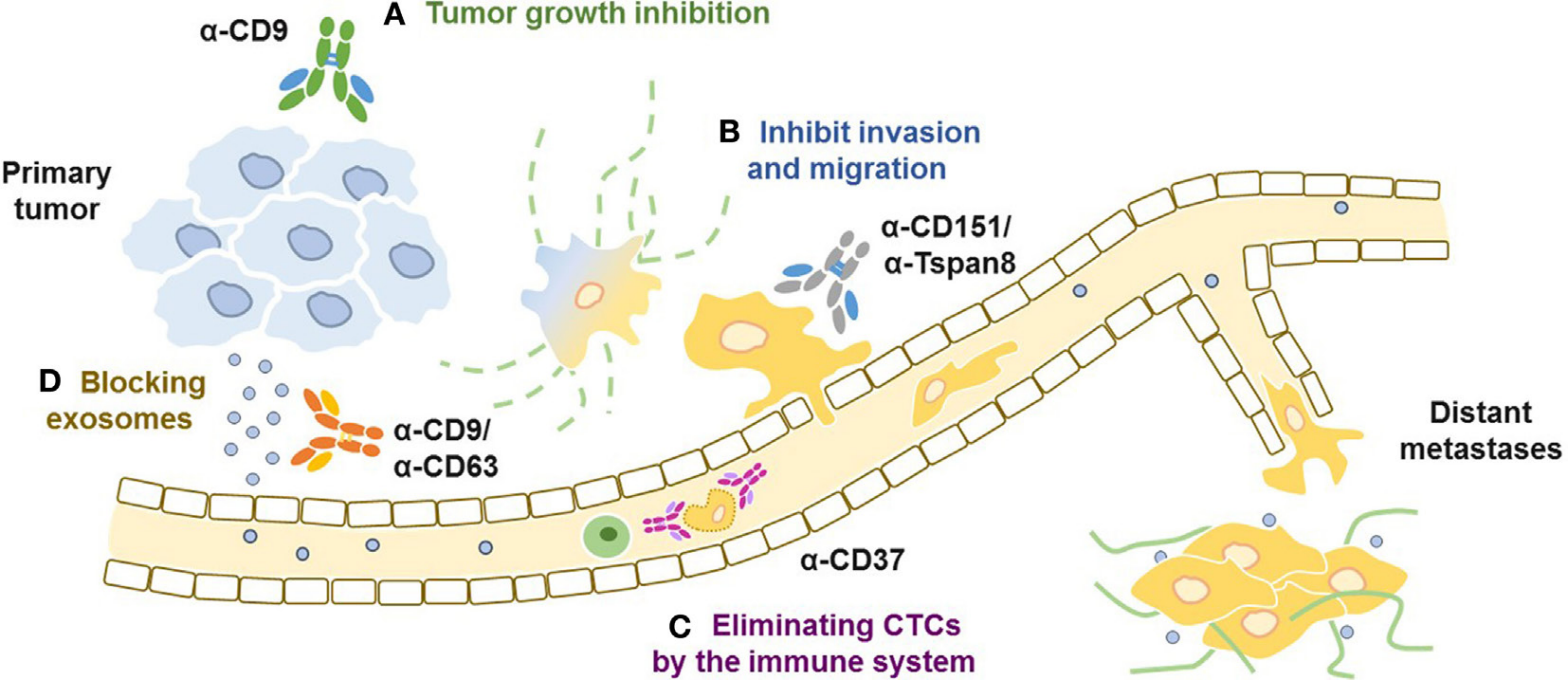
Targeting tetraspanins at different stages of tumor progression. **(A)** Anti-CD9 antibodies have been shown to inhibit proliferation of human gastric tumors **(B)** anti-Tspan8 and anti-CD151 antibodies which inhibit adhesion, migration, and extravasation of tumor cells in different types of cancer *in vitro* and *in vivo*. **(C)** Anti-CD37 antibodies eliminates circulating chronic lymphocytic leukemia cells by recruiting and activating the immune system, **(D)** anti-CD9 and anti-CD63 antibodies block exosomes and enhance their clearance from circulation through macrophage-dependent mechanisms.

At the cellular level, mAbs that target individual tetraspanin members have been used to study signaling pathways, to disrupt molecular associations, to analyze the dynamics of cell surface partitioning, and to probe the tetraspanin web (54). Because tetraspanins regulate cell adhesion, invasion, and metastases, a strategy that prevents any of these cell functions seems reasonable (Figure 2). Indeed, several anti-tetraspanins’ mAbs inhibit tumor cell invasion and migration (7, 55). However, not all antibodies that target the same individual tetraspanin share the same properties, suggesting that engagement of specific epitopes in the tetraspanin molecule could result in different outcomes. Thus, an anti-CD9 mAb (PAINS-13) that disrupts the association of CD9 with β1 integrin (56) inhibited the growth of a human colon carcinoma cell line xenograft more effectively than another anti-CD9 mAb (VJI/20) or integrin mAbs (57). Yet, all tested anti-CD9 mAbs (VJI/10, VJI/20, and GR2110) inhibited the trans-endothelial migration of melanoma cells (58). An additional anti-CD9 antibody (ABL6) (59) induced apoptosis (60) and reduced the growth of a human gastric cancer cell line in a xenograft model (61).

The disruption of the interaction with integrins has been studied even in more details for anti-CD151 mAbs whose epitope map to the QRD sequence in the large extracellular loop (LEL) (33). Anti-CD151 mAbs that disrupt the interaction with integrins prevent prostate cancer metastases, in contrast to anti-CD151 mAbs that bind to integrin-associated CD151 (62–64). Indeed, anti-CD151 mAb (1A5) blocked metastases in SCID mice bearing Hep-3 tumors by inhibiting invasion and migration, although the antibody did not inhibit primary tumor growth (65). However, a study using a different monoclonal antibody (SFA1-2B4) that co-immunoprecipitated CD151 with α3β1 integrin prevented lung metastases of human colon cancer and fibrosarcoma cell lines (66). TEMs in endothelial cell junctions include CD151, CD81, and CD9, a study comparing migration of endothelial cells demonstrated that anti-CD151 and CD81, as well as anti-integrin mAbs inhibited migration (67).

More recent studies have shown that mAb targeting Tspan8 (Ts29.2) inhibited the growth of two human colorectal cancer cell lines when injected into nude mice, interestingly the antibody did not induce direct toxicity nor the inhibition of the previously reported angiogenic properties of Tspan8 (68). Moreover, a mAb reacting with the LEL of Tspan8 inhibited tumor invasion *in vitro* and diminished incidence of ovarian metastases *in vivo* (26, 69). Recently, a radiolabeled anti-Tspan8 mAb, labeled with lutetium-177 was effective against colorectal cancer in a xenograft model, showing a significantly reduced tumor growth (70).

Taken together, anti-tetraspanin mAbs have shown significant anti-tumor effects *in vitro* and in mouse models, but because of expression in both tumor and host, off-target effects are still of major concerns. Strategies to reduce the risk of off-target effects could include the use of bispecific mAbs that target both the individual tetraspanin and its interacting partner, for example, CD81/CD19 in B cells.

## Exploiting Tetraspanin Function for Immunotherapy

Indeed, a bispecific antibody was engineered to target CD63 on one arm thereby enabling efficient internalization of an anti-HER2 arm that targets the tumor (71). This bispecific construct allows targeting a drug-conjugated tumor binding-antigen, HER2, to the lysosomal pathway by CD63. This novel approach resulted in an improved survival and delayed tumor growth in a xenograft model of ovarian cancer. Mice treated with the bispecific bsHER2xCD63-Duostatin-3 conjugate increased HER2 internalization, this effect was not observed with the monovalent antibodies targeting only HER2 or CD63. This interesting approach of exploiting CD63 for cancer immunotherapy is based on its role in intracellular trafficking and abundance in exosomes (72).

Targeting tetraspanins in exosomes for cargo delivery has been reviewed extensively (5, 73). Exosomes gained attention due to their important function during cellular communication, in addition to tetraspanins they contain a variety of different molecules; receptors, integrins, lipids, mRNAs, and miRNA, all of which have an effect in their target cells (74). Importantly exosomes released from cancer cells have been shown to support metastases. Thus, blocking exosomes from tumor cells with antibodies could be used as a therapeutic strategy to prevent metastases, as shown for cancer stem cells (75). Although tetraspanins (i.e., Tspan8, CD81, and CD63) were originally used as exosome markers it is now clear that they play an active role in exosome cargo-loading and delivery (5, 73). Proteomic analysis using tetraspanins c-terminal domains to pull-down interacting proteins showed a significant overlap with proteins found in exosomes, suggesting that tetraspanins might regulate protein cargo (76). In addition, it has been shown that several tetraspanins regulate protein trafficking to the membrane and intracellular compartments of several receptors (48, 49). Indeed, deleting CD81 revealed a differential protein cargo in exosomes lacking CD81 (76). Moreover, deleting CD81 in endothelial-producing exosome cells but not tetraspanins CD63 or CD82 reduced breast cancer motility and metastasis (77). It is, therefore, likely that individual tetraspanins might regulate protein loading in exosomes, and that such specificity could potentially be exploited to confer selective exosome cargo and/or delivery. For example, the preferential interaction of Tspan8 with α4β4 in exosomes (78). Such preferential interaction could render uptake-specificity of exosome by endothelial and pancreatic cells, and possibly facilitate the use of exosomes for drug delivery, reviewed in Ref. (79).

Proteomic profiling of extracellular vesicles of 60 cancer cell lines (NCI-60) revealed CD81 expression in all 60, while CD9 and CD63 were expressed in about 40 of these cell lines (80). Moreover, clinically relevant exosomes isolated with anti-CD9 or anti-CD63 antibodies and then detected with anti-HER2 revealed a 14–35% tumor-specific exosomes from breast cancer patient serums, which potentially could be used as non-invasive diagnostic method or even used to detect disease progression (81). A study that used anti-CD9 and anti-CD63 antibodies to deplete tumor-derived exosomes in a xenograft model showed a significant reduction in metastases to different organs but had no effect on growth of primary tumors (82). That study showed that exosome depletion from blood was macrophage-mediated. However, in a xenograft model only the tumor tetraspanins are targeted by the antibodies, whereas in human the targeted tetraspanins are expressed both in the host and in the tumors.

## From Bench to Bedside

Several tetraspanins used as therapeutic targets show promise in preclinical models of tumor progression and metastases (7, 28, 55, 83). However, CD37 is the only tetraspanin target that has moved forward into the clinic (84). CD37 is predominantly and abundantly expressed on mature B cell malignancies, but not on solid tumors. B cells serve as especially suitable targets for immunotherapy because of the ability of the antibodies to mediate both direct and indirect immune responses. This therapy is a promising tool, especially in those cases where other immunotherapies have failed. Different anti-CD37 antibodies that better mediate antibody-dependent cell cytotoxicity, improve complement activation, or are conjugated to a cytotoxic drugs (83), have recruited patients to clinical trials (clinicalTrials.gov).

## Concluding Remarks

Tetraspanins regulate cancer progression and metastases; yet, their broad tissue distribution presents an impediment for cancer immunotherapy, due to possible off-target effects. However, with current exponential advances in immunotherapy, these limitations could be overcome. Examples include bispecific antibodies that confer tumor selectivity or shielding antibodies with tumor-specific proteases (85). Lastly, understanding the function of tetraspanins and their molecular partners, both in the tumor and in the host, will ultimately develop new therapies for cancer treatment.

## Author Contributions

FVC ans SL reviewed literature and wrote the manuscript. FVC prepared the figures. All authors approved the manuscript for publication.

## Conflict of Interest Statement

The authors declare that the research was conducted in the absence of any commercial or financial relationships that could be construed as a potential conflict of interest. The handling Editor declared a past co-authorship with one of the authors SL.
